# A Novel Ratiometric Photoelectrochemical Biosensor Based on Front and Back Illumination for Sensitive and Accurate Glutathione Sensing

**DOI:** 10.3390/bios14060285

**Published:** 2024-06-01

**Authors:** Jie Huang, Florian Ion Tiberiu Petrescu, Bing Li, Likui Wang, Haiyan Zhu, Ying Li

**Affiliations:** 1 The Key Laboratory of Synthetic and Biotechnology Colloids, Ministry of Education, School of Chemical and Material Engineering, Jiangnan University, Wuxi 214122, China; 7220610017@stu.jiangnan.edu.cn (J.H.); l13275102209@163.com (B.L.); lkwang@jiangnan.edu.cn (L.W.); bbszhy1979@163.com (H.Z.); 2 Department of Mechanisms and Robots Theory, National University of Science and Technology Polytechnic Bucharest, 060042 Bucharest, Romania

**Keywords:** ratiometric detection, photoelectrochemical biosensor, glutathione

## Abstract

The ratiometric detection method has a strong attraction for photoelectrochemical bioanalysis due to its high reliability and real-time calibration. However, its implementation typically depends on the spatial resolution of equipment and the pairing of wavelength/potential with photoactive materials. In this paper, a novel ratiometric photoelectrochemical biosensor based on front and back illumination was prepared for the detection of glutathione (GSH). Unlike traditional ratio methods, this ratiometric biosensor does not require voltage and wavelength modulation, thereby avoiding potential crosstalk caused by voltage and wavelength modulation. Additionally, the formation of a heterojunction between mTiO_2_ and Ag_2_S is conducive to enhancing light absorption and promoting charge separation, thereby boosting the photocurrent signal. Apart from forming a heterojunction with TiO_2_, Ag_2_S also shows a specific affinity towards GSH, thus enhancing the selectivity of the mTiO_2_/Ag_2_S ratiometric photoelectrochemical biosensor. The results demonstrate that the ratiometric photoelectrochemical biosensor exhibits a good detection range and a low detection limit for GSH, while also possessing significant interference elimination capability. The GSH detection range is 0.01–10 mmol L^−1^ with a detection limit of 6.39 × 10^−3^ mmol·L^−1^. The relative standard deviation of 20 repeated detections is 0.664%. Impressively, the proposed novel ratiometric PEC biosensor demonstrates enviable universality, providing new insights for the design and construction of PEC ratiometric sensing platforms.

## 1. Introduction

GSH is a ubiquitous non-protein thiol found in mammalian cells, consisting of glutamic acid, cysteine, and glycine. It plays a crucial role in various physiological processes, including the maintenance of redox homeostasis, the transmission of biological information, and immune response [1,2]. Moreover, GSH is used as a biomarker for the early identification of many diseases, such as liver disease, Alzheimer’s disease, and cancer [3,4,5]. Therefore, it is of great significance for human health to detect changes in GSH levels with a simple, accurate, and sensitive method. In this regard, several analytical techniques have been developed for the detection of GSH, including high-performance liquid chromatography [6], photoelectrochemistry [7], fluorescence analysis [8,9], colorimetry [10,11], the electrochemical method [12,13], and the surface-enhanced Raman effect [14,15]. Among these techniques, the photoelectrochemical method has been widely used in the field of bioanalytic chemistry because of its low background signal, high sensitivity, ease of equipment miniaturization, and cost-effectiveness. Various nanomaterials have been utilized to construct glutathione photoelectrochemical sensing platforms. For instance, an Au NPs/ZnO NRs array/FTO electrode demonstrated a detection limit of 2.7 μM within a concentration range of 10–800 μM, with a sensitivity of 0.06336 [16]. Similarly, a Pt–IrO_2_/TiO_2_NTs/Ti electrode exhibited a detection limit of 0.8 μM and a sensitivity of 54.174 within the concentration range of 1–10 μM [17].

For typical photoelectrochemical (PEC) bioanalysis, quantitative detection refers to the process whereby a semiconductor is illuminated with light (excitation signal), resulting in the generation of charge carriers (electron–hole pairs). Subsequently, these charge carriers are captured by the electron acceptor or donor, causing the transfer of electrons to the electrode and generating a photocurrent signal (detection signal). However, in the traditional single-signal readout mode, even minor disturbances in the detection instrument and environment may result in unreliable results, particularly for the trace analysis of target substances in complex detection environments. To reduce the interference of external factors and improve the accuracy of trace analysis, ratiometric PEC sensors have been extensively developed [18,19,20]. Unlike traditional single-signal sensors, ratio sensors exhibit a dual-signal response mode, where the ratio of two signals is used as the output signal instead of the absolute value of a single signal. The ratio sensor platform is constructed in two modes. In one mode, the target analyte induces a significant change in one signal as the response signal, while the other signal remains relatively unchanged as the reference signal. In another mode, the target analyte induces significant changes in both signals, both of which can serve as either the response or reference signal. Ultimately, a ratio-type sensor is constructed based on the ratio of these two signals. By self-calibrating the two output signals, the interference of the microenvironment and the detection system can be corrected, thereby improving the overall accuracy of the analysis.

Ratiometric photoelectrochemical sensors can be classified into three types: the potential-resolved, the spatial-resolved and the wavelength-resolved ratiometric photoelectrochemical sensors [21,22,23]. The spatial-resolved technology relies on adjusting the light source irradiation position to generate different signals, which usually requires the different electrode areas to be modified with different materials and tags [24,25]. To avoid interference from adjacent electrode tag diffusion, different regions are divided by insulating and hydrophobic materials or individually connected to multichannel instruments using conductive wires. The fabrication process is complex, and the partition material occupies most of the space of the sensor, severely limiting the electrode density and making high-resolution measurements difficult. Importantly, multi-area modification reduces the repeatability of the sensor, resulting in potential errors. Wavelength-resolved technology relies on light-absorbing active materials under different wavelengths. To avoid signal interference caused by multiple materials absorbing the same wavelength simultaneously, multiple light-absorbing materials with non-overlapping absorption regions need to be selected. Finding corresponding light-active materials to achieve ratiometric photoelectrochemical determination is still a huge challenge [26]. On the other hand, different wavelengths of light require the combination of multiple filters, which may cause uneven photon efficiency and induce errors [27]. Potential-resolved technology requires the different electrode areas on the same substrate to be modified with different light-active materials. When the applied voltage changes, one material produces a photocurrent while the other material remains “silent”. The electrochemical signals of the two materials do not overlap with each other, and this type of method is limited by material screening. Moreover, most light-active materials rely on high positive and negative voltages to make them “silent”. High voltage inevitably brings a series of side effects, such as electrode passivation, baseline drift, and biological damage [28]. To address the challenges of constructing traditional ratio-based sensors, an electronic transfer tunnel distance modulation strategy has been developed, albeit still relying on wavelength modulation. Recently, the illumination direction control of the photoanode has been demonstrated to influence the electron diffusion distance, which indicates that the transfer ability of interface charge carriers can be controlled by changing the direction of incident light irradiation on the electrode [29,30].

In light of the aforementioned findings, a novel ratiometric photoelectrochemical sensor based on front and back illumination has been developed for the accurate detection of GSH. By leveraging the differential charge carrier transport capabilities between front and back illumination, a ratio-based photoelectrochemical strategy has been implemented, eliminating the need for a light source or potential modulation and overcoming limitations associated with specific photoactive materials, thus demonstrating greater universality. A TiO_2_/Ag_2_S heterojunction structure has been designed to enhance photocurrent response and widen the disparity in charge carrier transport capabilities between front and back illumination configurations. Through the ratio determination of front-to-back illumination photocurrent signals (I_F_/I_B_) for GSH detection, significant improvements in linear curve correlation and reduction in relative standard deviation have been achieved. This study not only offers a facile extension to the determination of other analytes but also expands the diversity in the design of ratiometric sensing strategies.

## 2. Materials and Methods

### 2.1. Chemicals and Materials

Acetone (CH_3_COCH_3_), chloroform (CHCl_3_), ethanol (CH_3_CH_2_OH), methanol (CH_3_OH), hydrochloric acid (HCl), n-butyl titanate (TBOT), silver nitrate (AgNO_3_), sodium chloride (NaCl), potassium chloride (KCl), l-Arginine hydrochloride (C_6_H_14_N_4_O_2_), l-Glutamic acid (C_5_H_9_NO_4_) and glycine (C_2_H_5_NO_2_) were purchased from Sinopharm Chemical Reagent Co., Ltd., Shanghai, China. Pluronic F127 (F127) was obtained from Sigma Aldrich Trading Co., Ltd., Shanghai, China. Sodium sulfide nonahydrate (Na_2_S·9H_2_O), glutathione (GSH) and tris (hydroxymethyl) aminomethane were purchased from Shanghai Aladdin Biochemical Technology Co., Ltd., Shanghai, China. Ultrapure water was prepared by April UPW-30UV ultrapure water machine. Fluorine-doped Tin Oxide (FTO)conductive glass (thickness 2.2 mm, resistance 14 Ω) was procured from Wuhan Lattice Solar Technology Co., Ltd., Wuhan, China. All chemicals were used as received without further purification.

### 2.2. Apparatus

The surface morphologies of different samples were observed using a field emission scanning electron microscope (SEM, S-4800, Hitachi, Tokyo, Japan) equipped with an energy-dispersive X-ray spectrometer (EDS). X-ray diffractometer (XRD, Bruker AXS D8, Karlsruhe, Germany) was employed to explore the crystal structures of different samples. A fiber optic spectrometer (PG2000 pro, Shanghai Ideaoptics Company, Shanghai, China) was used to record the absorption spectra of the samples. The photoelectrochemical performance analysis was conducted in an electrochemical workstation (CHI660, Shanghai Chenhua Company, Shanghai, China), with a xenon lamp (Meiruichen MC-PF300C, Beijing, China) as the excitation light source. The simulated solar irradiation intensity was adjusted using a radiometer (CEL-FZ-A, China Education Au-light, Beijing, China). The standard three-electrode system was adopted for testing. The modified FTO, Ag/AgCl electrodes, and platinum net were working electrodes, reference electrodes, and pair electrodes, respectively.

### 2.3. Preparation of Initial Sols for F127-TiO_2_

Tetrabutyl titanate was dissolved and stabilized in HCl aqueous solution through intense stirring at room temperature. After 15 min, the titanium dioxide precursor solution was slowly added drop by drop to the ethanol solution containing F127. The molar ratio of each component in titanium dioxide sol was as follows: TBOT:F127:HCl:H_2_O:EtOH = 1:0.005:1.7:10:24. Subsequently, the TiO_2_ sol was stirred at room temperature and allowed to age for 3 h [31]. The aged TiO_2_ sol was then stored at a low temperature.

### 2.4. Preparation of mTiO_2_ Thin Films

The FTO conductive glass substrate (2 cm × 2 cm) was cleaned with acetone, chloroform, ethanol, and water in sequence by ultrasonication for 10 min to ensure surface cleanliness. Then, F127-TiO_2_ sol was spin-coated onto the cleaned FTO surface, with 100 μL of F127-TiO_2_ sol applied onto the FTO conductive side, and spun for 30 s at 3000 rpm. After repeating the spin-coating process 4 times, the sample was annealed at 450 °C for 1 h at a heating rate of 5 °C/min in an air atmosphere. The sample prepared using TiO_2_ sol containing F127 was named mTiO_2_, while the sample prepared using TiO_2_ sol without F127 was named TiO_2_.

### 2.5. Fabrication of mTiO_2_/Ag_2_S

The mTiO_2_ sample was first immersed in 0.1 M AgNO_3_ solution for 30 s, rinsed with deionized water, and thereafter annealed at 65 °C for 3 min. Next, the sample was immersed in 0.1 M Na_2_S solution for 30 s to allow a displacement reaction with sulfur, followed by rinsing with methanol, and finally annealed at 65 °C for 1 min. The immersion steps were repeated 5 times. Finally, the sample was heated to 400 °C at a heating rate of 10 °C/min in nitrogen and then annealed for 30 min.

### 2.6. Photoelectrochemical Measurements

A three-electrode system was employed for the detection of GSH, with a modified FTO serving as the working electrode, a saturated Ag/AgCl electrode as the reference electrode, and a Pt electrode as the counter electrode. The actual surface area of the working electrode was determined by fixing it with epoxy resin, and an optical photograph containing the electrodes and a ruler was captured using a digital camera. Subsequently, the optical photograph of the electrodes and ruler was imported into ImageJ software (imagej 1.52 h) for processing and analysis to determine the actual area. The PEC measurement was performed using I-T curve testing, with a 0.1 M Tris-HCl buffer solution (pH = 7.4) containing 0.1 M sodium chloride and 0.05 M potassium chloride serving as the electrolyte. Xenon lamps, with a wavelength range spanning from 300 nm to 2500 nm, serve as the excitation light source. The incident light intensity was adjusted to 100 mW/cm^2^ using a radiometer. A 0 V (V vs. Ag/Agcl) bias voltage was applied during all photoelectrochemical measurements. Electrochemical impedance spectroscopy (EIS) was performed under illumination conditions in the frequency range of 0.1 Hz to 100 kHz.

## 3. Results and Discussion

### 3.1. Characterization of the Materials

**Figure 1:**
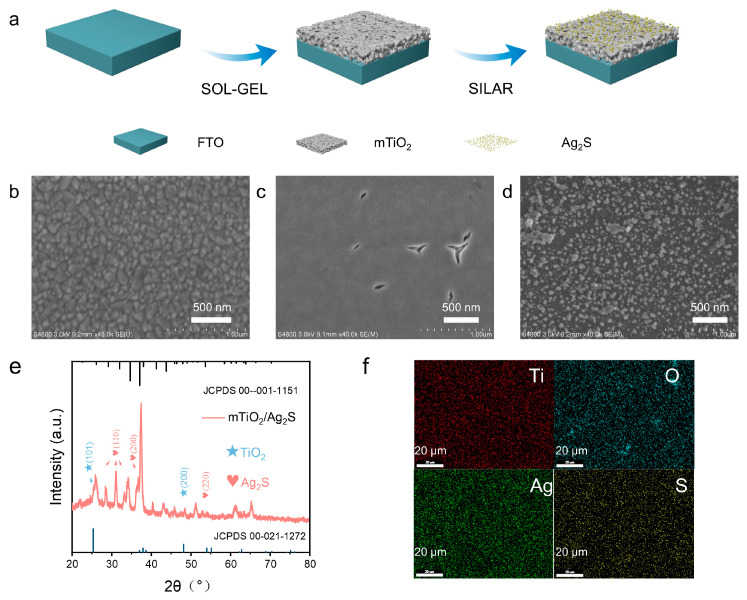
(**a**) Schematic diagram of the mTiO_2_/Ag_2_S Biosensor. SEM images of (**b**) FTO, (**c**) mTiO_2_ and (**d**) mTiO_2_/Ag_2_S. (**e**) X-ray diffraction (XRD) pattern of mTiO_2_/Ag_2_S. (**f**) EDS element mapping of Ti, O, Ag, and S in mTiO_2_/Ag_2_S.

The preparation process of mTiO_2_/Ag_2_S is illustrated in Figure 1a. Firstly, a TiO_2_ sol containing F127 is spin-coated onto an FTO substrate, followed by calcination to obtain mesoporous TiO_2_ film, which is attributed to the decomposition of F127 during calcination resulting in mesoporous structure formation [31]. As shown in Figure 1b,c, compared to FTO, the surface of mTiO_2_/FTO is denser and smoother, indicating the successful loading of mTiO_2_ film on the FTO surface. The mesoporous structure of mTiO_2_ is not only beneficial for the adsorption of photosensitizer Ag_2_S but also reduces the transfer distance of photo-generated charge carriers, facilitating the suppression of electron–hole recombination. Subsequently, the Ag_2_S film is prepared on the surface of mTiO_2_ by the successive ionic layer adsorption and reaction (SILAR) method. As depicted in Figure 1d, Ag_2_S nanoparticles with diameters of 50–200 nm are observed distributed on the mTiO_2_ surface. To further investigate the crystal phase structure of mTiO_2_/Ag_2_S, X-ray diffraction (XRD) characterization was performed. Figure 1e shows that the XRD pattern of mTiO_2_/Ag_2_S exhibits sharp and intense diffraction peaks, indicating good crystallinity. According to the JCPDS 00-021-1272 card, the diffraction peaks at 25.8° and 48.5° are attributed to the (101) and (200) planes of rutile phase TiO_2_, while according to the JCPDS 00-001-1151 card, the peaks at 28.5°, 31.1°, and 34.4° are ascribed to the monoclinic phase Ag_2_S (110) plane, and the peaks at 36.7° and 52.9° are attributed to the monoclinic phase Ag_2_S (200) and (220) planes, respectively [32,33]. These results demonstrate the successful preparation of mTiO_2_/Ag_2_S. Energy-dispersive X-ray spectroscopy (EDS) analysis in Figure 1f confirms the uniform distribution of Ti, O, Ag, and S on the substrate surface, providing further evidence for the successful preparation of mTiO_2_/Ag_2_S.

### 3.2. PEC Performance

**Figure 2:**
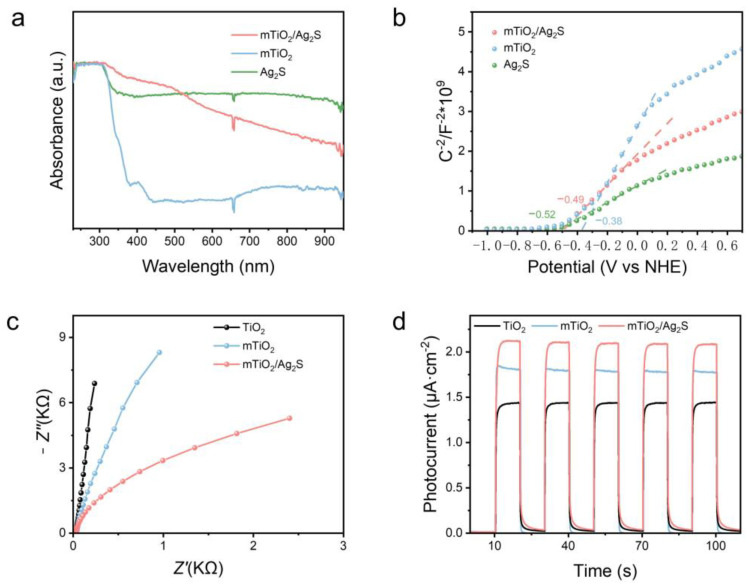
(**a**) UV–V is absorption spectra and (**b**) Mott–Schottky plots of Ag_2_S, mTiO_2_, and mTiO_2_/Ag_2_S. (**c**) EIS Nyquist plots and (**d**) photocurrent responses of TiO_2_, mTiO_2_, and mTiO_2_/Ag_2_S.

The photoelectrochemical properties play a crucial role in the sensing performance of sensors. Therefore, the influence of mTiO_2_/Ag_2_S heterojunction formation on the photoelectric performance was investigated. The UV-visible absorption spectra in Figure 2a depict the light-capturing abilities of Ag_2_S, mTiO_2_ and mTiO_2_/Ag_2_S. It can be observed that TiO_2_ exhibits significant absorption capability in the ultraviolet region, with an absorption edge at approximately 390 nanometers, while Ag_2_S demonstrates remarkable absorption in both the ultraviolet and visible light regions [34,35]. In contrast, upon Ag_2_S modification on the surface of mTiO_2_, there is a clear absorption in the visible light region. This is attributed to the strong visible light absorption of Ag_2_S and the effective improvement of the photogenerated carrier generation and separation by the formation of Type-II heterojunction between Ag_2_S and mTiO_2_ [36]. To validate the promotion of electron–hole pair separation by the mTiO_2_/Ag_2_S heterojunction, Mott–Schottky tests were performed on the mTiO_2_, Ag_2_S, and mTiO_2_/Ag_2_S samples. In Figure 2b, both mTiO_2_ and Ag_2_S display positive-slope N-type semiconductor characteristics, with flat band potentials (E_fb_) of −0.38 and −0.52 V (V vs. NHE), respectively. E_fb_ serves as an indicator of the internal field strength that drives charge transfer at the semiconductor/electrolyte interface. A higher E_fb_ value indicates a higher chemical potential gradient formed from the semiconductor to the electrolyte, thereby ensuring rapid charge transfer at the semiconductor/electrolyte interface [37]. Compared to mTiO_2_, mTiO_2_/Ag_2_S demonstrates a higher E_fb_ value, which confirms that the Type-II heterojunction between mTiO_2_ and Ag_2_S promotes rapid charge transfer at the interface. This conclusion is also supported by EIS Nyquist plots, where the diameter of the semicircle is directly related to the interface charge transfer resistance (R_ct_). A smaller semicircle diameter indicates lower electrode charge transfer resistance. As shown in Figure 2c, the R_ct_ of mTiO_2_ is lower than that of TiO_2_, possibly due to the porous structure providing abundant reaction sites or enhanced light absorption. A significantly reduced R_ct_ is observed for mTiO_2_/Ag_2_S compared to mTiO_2_. To further confirm the significantly improved photoelectrochemical performance of the mTiO_2_/Ag_2_S composite material compared to TiO_2_, the PEC activity of TiO_2_, mTiO_2_, and mTiO_2_/Ag_2_S photoelectrodes was tested through I-t experiments. According to Figure 2d, the photocurrent signal of wide bandgap mTiO_2_ is relatively low due to its limited light absorption range under illumination. However, after Ag_2_S modification, the photocurrent signal is significantly enhanced. These results validate that the mesoporous TiO_2_ and the mTiO_2_/Ag_2_S Type-II heterojunction structure effectively enhance light absorption and suppress electron–hole pair recombination, thereby improving the photoelectrochemical performance.

### 3.3. PEC Ratio Sensing Mechanism of GSH

**Figure 3:**
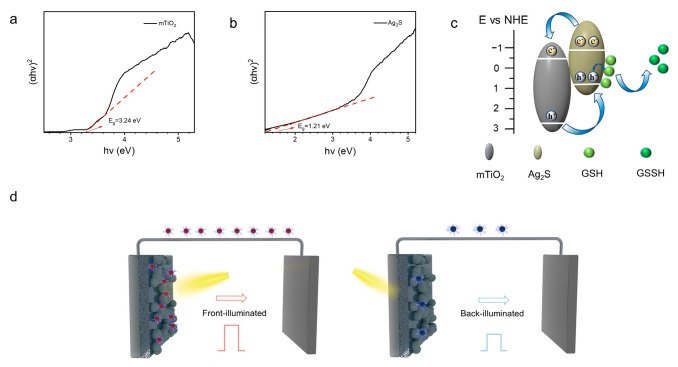
Band gap values of (**a**) mTiO_2_ and (**b**) Ag_2_S. (**c**) The mechanism illustration of the mTiO_2_/Ag_2_S PEC sensor for GSH detection. (**d**) The ratiometric PEC sensing mechanism.

To investigate the sensing mechanism of the TiO_2_/Ag_2_S ratiometric photoelectrochemical sensor, the band gaps, conduction band positions, and valence band positions of mTiO_2_ and Ag_2_S need to be determined. The bandgaps (E_g_) of mTiO_2_ and Ag_2_S semiconductors were calculated using the Tauc equation:

αhν = A(hν − E_g_)^n/2^ (1)

where α is the absorption coefficient, h is the Planck constant, ν is the light frequency, A is the proportionality constant, E_g_ is the band gap, and n is decided by the transition type of the semiconductor [38]. As shown in Figure 3a,b, the bandgaps of mTiO_2_ and Ag_2_S are 3.24 eV and 1.21 eV, respectively. In the N-type semiconductor, the gap between the conduction band edge and the flat band edge is approximately 0.1 V [39]. The valence band positions were estimated according to the following formula:

E_VB_ = E_CB_ + E_g_ (2)

The E_VB_ represents the conduction band potential, the E_CB_ is the valence band potential, and the E_fb_ is the flat band potential. The E_fb_ values of mTiO_2_ and Ag_2_S are determined to be −0.38 V and −0.52 V (vs. NHE), respectively, based on the Mott–Schottky (M-S) curves shown in Figure 2b. The valence band positions of mTiO_2_ and Ag_2_S are calculated to be 2.76 V and 0.59 V (vs. NHE), respectively, according to Equation (2). Building upon these results, a potential detection mechanism of GSH is established using the mTiO_2_/Ag_2_S PEC sensor, as illustrated in Figure 3c. The mTiO_2_/Ag_2_S system forms a type-II heterojunction, which broadens the light absorption range and significantly enhances the efficiency of photo-induced charge carrier separation. Under illumination, the two photoactive materials (mTiO_2_ and Ag_2_S) are excited, with electrons in the valence band of mTiO_2_ and Ag_2_S being excited to the conduction band, therefore generating holes in the valence band. Due to the formation of the heterojunction, photogenerated electrons rapidly transfer from the conduction band of Ag_2_S to the conduction band of TiO_2_ through the heterojunction structure, subsequently transferring to the external circuit. Meanwhile, photogenerated holes transfer from the valence band of TiO_2_ to the valence band of Ag_2_S. The matching of band structures results in the formation of an internal electric field, leading to enhanced separation of electrons and holes. The PEC response mechanism for GSH is based on the acceleration of charge transfer by the electron donor. On one hand, Ag_2_S exhibits a specific affinity for GSH-containing thiol groups. On the other hand, GSH acts as a scavenger for photogenerated holes, effectively reducing the electron–hole recombination rate and thereby amplifying the photocurrent response. Figure 3d illustrates the principle behind the construction of the ratio-type PEC sensing platform. When incident light illuminates the front side of the electrode (the left part of Figure 3d), both TiO_2_ and Ag_2_S can be simultaneously excited. The formation of a heterojunction between TiO_2_ and Ag_2_S greatly enhances the efficiency of charge separation, resulting in a relatively strong output of the photoelectric current signal from the photoelectrode. However, when incident light irradiates the back side of the electrode (the right part of Figure 3d), several factors come into play. Firstly, due to the strong absorption capability of FTO in the ultraviolet region, a portion of the light is absorbed by FTO, leading to a reduction in the light absorption of the photoactive material. Additionally, the FTO and TiO_2_ layers possess certain thicknesses, which impede the transmission of light to Ag_2_S. These dual factors collectively result in a decreased light absorption efficiency of the photoelectrode, consequently yielding a relatively weaker output of the photoelectric current signal. Therefore, a novel ratiometric PEC sensor is developed for the quantitative detection of GSH based on the different photocurrent responses generated by the front and back illumination of GSH.

### 3.4. Optimization of Experimental Conditions

**Figure 4:**
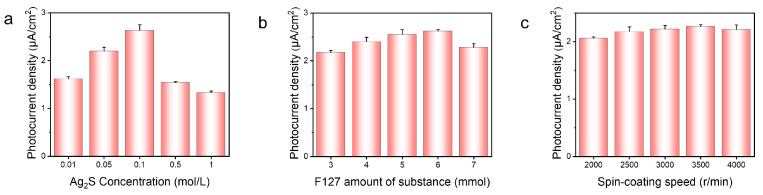
The photocurrent intensity varies with (**a**) Ag_2_S concentration, (**b**) F127 amount of substance, and (**c**) TiO_2_ spin-coating speeds.

To enhance the performance of the PEC sensor for detecting GSH, experimental conditions were optimized for the concentrations of Ag_2_S, the molar mass of F127, and the spin coating speeds of TiO_2_ sol. All PEC tests of the optimization process were performed under a light intensity of 100 mW/cm^2^ and a 0 V bias voltage. The optimal conditions were determined based on the photocurrent intensity value of mTiO_2_/Ag_2_S. In Figure 4a, the effect of varying Ag_2_S concentration was studied. The photocurrent intensity initially increases and then decreases with increasing Ag_2_S concentration. Notably, the photocurrent reaches its peak when the Ag_2_S concentration is 0.1 M. The reduction in photocurrent intensity observed at high concentrations of Ag_2_S may potentially be attributed to the agglomeration phenomenon of Ag_2_S. As shown in Figure 4b, the photocurrent response of the prepared photoelectrode is maximized when the amount of F127 added to the TiO_2_ sol is 5 mmol. The introduction of the F127 pore-forming agent increases the specific surface area of TiO_2_ and provides more binding sites for Ag_2_S loading. This favors the formation of more heterojunctions, which in turn accelerate charge transfer and enhance light absorption. However, excessive F127 may result in too many surface defects acting as composite centers, which increase the electron–hole recombination efficiency and possibly cause a decrease in photocurrent signal [40]. The photocurrent intensity is impacted by the spin-coating speeds, as it affects the uniformity and loading amount of TiO_2_ on the FTO surface. At low spin-coating speeds, an excessive loading of TiO_2_ causes TiO_2_ accumulation, producing an increasing number of surface composite centers. As the surface composite centers proliferate, the diffusion resistance of electron motion increases, resulting in a decrease in photocurrent intensity. However, higher spin-coating speeds also contribute to a decrease in photocurrent, as the lower loading amount of TiO_2_ leads to overall insufficient light absorption. As depicted in Figure 4c, the photocurrent initially increases and then decreases with the spin-coating speeds varying, with the maximum photocurrent observed at a spin-coating speed of 3500 rpm.

### 3.5. Assay Performance of the PEC Sensor

**Figure 5:**
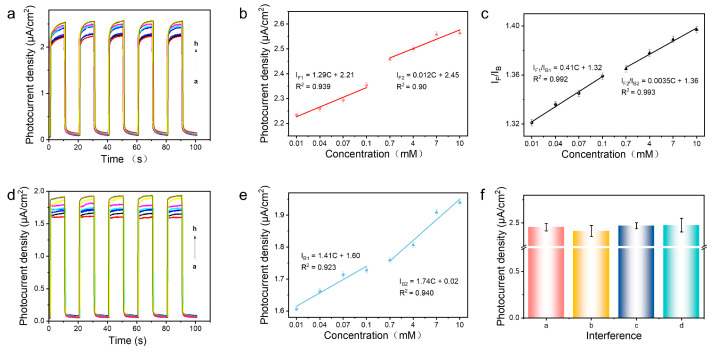
(**a**) Front illumination photocurrent responses (I_F_) for different concentrations of GSH (a→h: 0.01→10 mM) and (**b**) corresponding calibration curves. (**d**) Back illumination photocurrent responses (I_B_) for different concentrations of GSH (a→h: 0.01→10 mM) and (**e**) corresponding calibration curves. (**c**) Linear calibration curves between I_F_/I_B_ and concentrations of GSH (a→h: 0.01→10 mM). (**f**) Anti-interference capability of the proposed PEC sensor ((a: 1 mM GSH, b: 1 mM GSH + 5 mM L-arginine, c: 1 mM GSH + 5 mM L-glutamic acid, d: 1mM GSH + 5 mM L-glycine).

**Figure 6:**
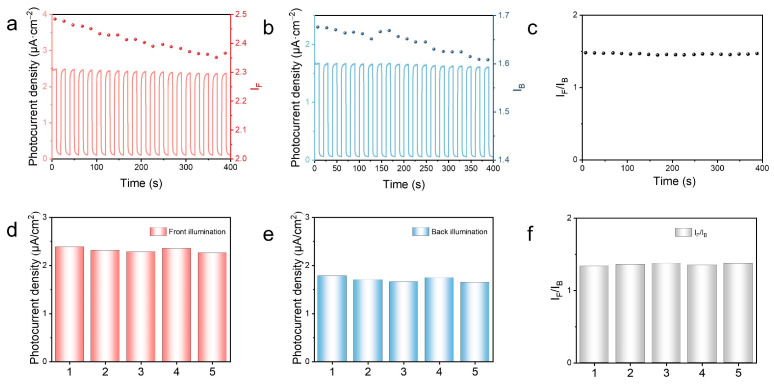
(**a**) Front illumination and (**b**) back illumination photocurrent stability of the mTiO_2_/Ag_2_S-based PEC biosensor in the electrolyte containing 0.1 mM of GSH under intermittent visible light irradiation for 400 s (**c**) 20 times photocurrent ratio (I_F_/I_B_). (**d**) Front illumination and (**e**) back illumination photocurrent signal of five parallel photoelectrodes in the electrolyte containing 0.1 mM of GSH. (**f**) Five parallel photoelectrode signal ratio (I_F_/I_B_).

Under the aforementioned optimized conditions, the analytical performance of the mTiO_2_/Ag_2_S photoelectrochemical sensor for GSH was evaluated by measuring the I-T curves of the photoelectrode with different concentrations of GSH in 0.1 mM Tris (pH = 7.4) solution. Figure 5a,d show the variation curves of front illumination photocurrent (I_F_) and back illumination photocurrent (I_B_) when 0.01–10 mM GSH was added to 0.1 M Tris (pH = 7.4) solution. Observations reveal that both I_F_ and I_B_ witness a marked increase with the rise in GSH concentration, with I_F_ being greater than I_B_ at any given concentration of GSH. Such findings provide supporting evidence for the validity of the mTiO_2_/Ag_2_S ratiometric photoelectrochemical sensor’s proposed design. Figure 5b,e present the linear fitting curves for both I_F_ and I_B_ concerning GSH concentration. A positive correlation between photocurrent density and GSH concentration can be observed. Furthermore, within the GSH concentration range of 0.01–10 mM, two-segment linear relationships are exhibited between GSH concentration and both I_F_ and I_B_. When the concentration of glutathione is at a low water level, the local concentration on the electrode surface is rapidly depleted as glutathione is converted into a product, resulting in a high sensitivity of the photocurrent response. When the concentration of glutathione is high, diffusion of glutathione is hindered, resulting in decreased sensitivity [40]. Under front illumination conditions, two linear regression equations are derived as follows: I_F1_ = 1.29C + 2.21 (R^2^ = 0.939) and I_F2_ = 0.012C + 2.45 (R^2^ = 0.900), with a detection limit (LOD) of 9.33 × 10^−3^ mmol·L^−1^ (S/N = 3). Under the back illumination condition, the two linear regression equations are I_B1_ = 1.41C + 1.60 (R^2^ = 0.923) and I_B2_ = 1.74C + 0.02 (R^2^ = 0.940), with a LOD of 8.01 × 10^−3^ mmol·L^−1^. The above results indicate that both front and back illumination photocurrent responses exhibit low detection limits for GSH sensing, with the latter having lower detection limits due to its lower background signal compared to the former [41]. It is worth noting that the linear relationships between GSH concentration and both I_F_ and I_B_ are poor and can not satisfy the accurate measurement of GSH. The dual photocurrent signals obtained from front-/back-illuminated electrodes can be ratioed, which provides self-calibration and improves the reliability of the results. A strong linear relationship between I_F_/I_B_ and GSH concentration is observed, as shown in Figure 6c. Two linear regression equations were derived, namely I_F1_/I_B1_ = 0.41C + 1.32 (R^2^ = 0.992) and I_F2_/I_B2_ = 0.0035C + 1.36 (R^2^ = 0.9931). The detection limit is determined to be 6.39 × 10^−3^ mmol·L^−1^. These results indicate that the ratio-based sensor displays excellent linear correlation with GSH concentration, thereby effectively reducing errors and achieving satisfactory accuracy in biosensing applications. To evaluate the performance of the sensing system, the anti-interference capability of GSH detection was investigated by the biosensor. 0.1 mM GSH solution was prepared, and common interfering substances including glycine, glutamic acid, and arginine were added at a concentration of 0.5 mM for detection. No significant change in photocurrent was observed, as depicted in Figure 6f, indicating the excellent anti-interference ability of the mTiO_2_/Ag_2_S-based photoelectrochemical sensing system. The stability of the photoelectrode is also an important parameter for the sensor. To assess the stability of the mTiO_2_/Ag_2_S ratio-based photoelectrochemical sensor, the mTiO_2_/Ag_2_S electrode was subject to chopped light irradiation for 400 s in a Tris solution (pH = 7.4) containing 0.1 mM GSH. It can be seen from Figure 6a,b that both I_F_ and I_B_ exhibit stable and reproducible photocurrents. Additionally, Figure 6c displays the photocurrent values of I_F_ (a) and I_B_ (b) from 20 chopped light cycles, with the corresponding relative standard deviations (RSD) calculated as 1.683% and 1.377%, respectively. Importantly, the RSD of I_F_/I_B_ calculated from 20 chopped light cycles is 0.664%, significantly lower than that of the individual signals I_F_ and I_B_. The repeatability of the sensor is also a crucial parameter. As illustrated in Figure 6d,e, the comparison of photocurrent signals from five parallel electrodes reveals that the signals remain relatively consistent, indicating good reproducibility of the sensor. Figure 6f presents the I_F_/I_B_ values of the five parallel electrodes, with a calculated RSD of 1.135%. This RSD value is lower than the RSD of the front illumination photocurrent signal (2.192%) and the back illumination photocurrent signal (3.316%). These results collectively highlight the superiority of the ratio-based sensor.

## 4. Conclusions

In summary, a novel ratiometric PEC sensor has been successfully established for GSH detection based on the strategy of modulating the charge carrier transport capabilities through front and back illumination. By modifying the surface of mTiO_2_ with Ag_2_S to construct a type-II heterojunction, the photocurrent signal has been amplified to enhance the sensitivity of the sensor. Additionally, Ag_2_S provides specific recognition sites for detecting GSH, significantly improving the sensor’s selectivity. Experimental results demonstrate that the ratiometric mTiO_2_/Ag_2_S sensor exhibits precise and sensitive detection capabilities for GSH concentrations within the range of 0.01–10 mmol L^−1^. Importantly, the newly developed ratiometric photoelectrochemical sensing platform can be easily extended to the determination of other analytes, providing a fresh perspective for the design of ratiometric sensors.

## Data Availability

The data presented in this study are available on request from the corresponding author.
